# Application Techniques and Concentrations of Ascorbic Acid to Reduce Saline Stress in Passion Fruit

**DOI:** 10.3390/plants13192718

**Published:** 2024-09-28

**Authors:** Edmilson Júnio Medeiros Caetano, André Alisson Rodrigues da Silva, Geovani Soares de Lima, Carlos Alberto Vieira de Azevedo, Luana Lucas de Sá Almeida Veloso, Thiago Filipe de Lima Arruda, Allesson Ramos de Souza, Lauriane Almeida dos Anjos Soares, Hans Raj Gheyi, Mirandy dos Santos Dias, Lucyelly Dâmela Araújo Borborema, Vitória Dantas de Sousa, Pedro Dantas Fernandes

**Affiliations:** 1 Academic Unit of Agricultural Engineering, Federal University of Campina Grande, Campina Grande 58430-380, PB, Brazil; edmilson.junio@estudante.ufcg.edu.br (E.J.M.C.); carlos.azevedo@professor.ufcg.edu.br (C.A.V.d.A.); luana.lucas@estudante.ufcg.edu.br (L.L.d.S.A.V.); filipe@estudante.ufcg.edu.br (T.F.d.L.A.); allesson.ramos@estudante.ufcg.edu.br (A.R.d.S.); hans.gheyi@ufcg.edu.br (H.R.G.); mirandy.santos@estudante.ufcg.edu.br (M.d.S.D.); lucyelly.damela@estudante.ufcg.edu.br (L.D.A.B.); vitoria.dantas@estudante.ufcg.edu.br (V.D.d.S.); pedro.dantas@professor.ufcg.edu.br (P.D.F.); 2 Academic Unit of Agrarian Sciences, Federal University of Campina Grande, Pombal 58840-000, PB, Brazil; lauriane.almeida@professor.edu.br

**Keywords:** fruit growing, non-enzymatic compound, *Passiflora edulis* sims, salt stress

## Abstract

Salinity restricts the growth of irrigated fruit crops in semi-arid areas, making it crucial to find ways to reduce salt stress. One effective strategy is using eliciting substances like ascorbic acid. In this context, the objective of this study was to evaluate the effects of application methods and concentrations of ascorbic acid on the morphophysiology and production of sour passion fruit irrigated with saline water. The experiment was organized using a factorial randomized block design (3 × 3 × 2) with three application methods (soaking, spraying, and soaking and spraying), three concentrations of ascorbic acid (0, 0.8, and 1.6 mM) and two levels of electrical conductivity of irrigation water—ECw (0.8 and 3.8 dS m^−1^). Foliar spraying of ascorbic acid at a concentration of 0.8 mM mitigated the effects of salt stress on the relative water content of leaves, the synthesis of photosynthetic pigments, gas exchange, and total production of sour passion fruit when irrigated with ECw of 3.8 dS m^−1^. Plants grown with water of 0.8 dS m^−1^ and under foliar application of 0.8 mM of ascorbic acid achieved the maximum growth in stem diameter and the greatest volume of pulp in the fruits.

## 1. Introduction

Belonging to the Passifloraceae family, sour passion fruit (*Passiflora edulis* Sims) is a fruit crop grown in tropical and subtropical countries. In 2022, Brazil produced 697,859 tons in an area of 45,602 hectares, with the Northeast having the largest planted area, however, the average productivity in this region is only 14,765 kg per hectare, which is lower than the yield in the southern region of the country (21,290 kg per hectare) [1].

From an economic and commercial point of view, the cultivation of sour passion fruit plays a role of considerable importance in Brazil, since this species has a significant impact on the generation of employment and income throughout the year, especially for small- and medium-sized producers living in the semi-arid regions of Brazil [2].

The occurrence of irregular rainfall throughout the year, combined with intense evaporation, favors the accumulation of salts in the water sources of this region. This increase in salinity generates negative impacts on plants, leading to restrictions in the absorption of water and nutrients due to osmotic and ionic stress, thus affecting physiological processes, growth, and production components [3,4].

Using water with high concentrations of salts in irrigation can impair the metabolic and biochemical functions of salt stress-sensitive plants such as sour passion fruit, resulting in the suppression of their production potential through the partial closure of stomata, reductions in photosynthesis, protein synthesis and enzymatic activities, and the degradation of photosynthetic pigments [5]. Salinity also has the potential to modify electron transport, affecting the activity of photosystem II, which plays a crucial role in the oxidation of water molecules to generate electrons [6], resulting in changes in oxidative homeostasis.

Recent studies have shown that irrigation with saline water can negatively affect passion fruit cultivation; for example, Lima et al. [7] highlighted that irrigation with water of ECw of 0.3 dS m^−1^ or higher reduces the relative water content, gas exchange, number of fruits, and production per plant, and increases electrolyte leakage in the leaf blades of passion fruit plants. Galvão Sobrinho et al. [8] evaluated passion fruit production and obtained a threshold water salinity of 0.8 dS m^−1^, with a reduction of 14.78% per unit increase in ECw above this level. Andrade et al. [9] highlighted that water salinity above 0.7 dS m^−1^ reduces the physical and chemical quality of passion fruit.

Several strategies have been adopted to reduce the effects of salt stress on plants, including the application of ascorbic acid (AsA) [10]. AsA can reduce the excessive generation of reactive oxygen species (ROS) by collaborating with the enzyme ascorbate peroxidase (APX), aiding plant acclimatization during exposure to salinity [11]. AsA also acts to protect proteins and lipids from oxidative stressors such as salinity and drought. This compound contributes to plant growth, photosynthesis rate, transpiration, oxidative defense potential, and photochemical pigments, which can improve tolerance to abiotic stresses [10,12]

In the literature, there are reports on the application of AsA as an alternative to reduce the adverse effects of salt stress on different crops such as radish [13], cowpea [14], barley [15], and sugar beet [16]. However, studies addressing the effects of application methods and concentrations of AsA in fruit species such as sour passion fruit subjected to irrigation with brackish water under semi-arid conditions are incipient.

Considering the importance of this fruit crop in the social and economic context of agribusiness, it is essential to develop strategies capable of mitigating the effects of salt stress on plants and enabling the expansion of irrigated agriculture in semi-arid regions. In this context, this study is based on the hypothesis that the deleterious effects caused by salt stress on the growth, physiology, and production of passion fruit can be mitigated by the use of AsA through the concentration and application method most appropriate for the crop.

In this context, the objective of this study was to evaluate the effects of application methods and concentrations of AsA on the morphophysiology and production of sour passion fruit irrigated with saline waters in a semi-arid area.

## 2. Results

The original multidimensional space of variables was condensed into two principal components (PC1 and PC2) with eigenvalues of λ ≥ 1.0 [17]. These components, as detailed in Table 1, accounted for 86.1% of the total variance. PC1, which explained 67.52% of the variance, included most of the variables except for average fruit weight (AFW), fruit polar diameter (FPD), and fruit equatorial diameter (FED). PC2 accounted for 18.58% of the remaining variance and comprised the variables AFW, FPD, and FED.

The interaction between application methods, AsA concentrations, and electrical conductivity levels of irrigation water (ECw) had a significant effect (*p* ≤ 0.05) on the two principal components (Table 1). Additionally, each individual factor significantly affected all variables (*p* ≤ 0.01).

The analysis of the correlations between the variables (Table 2) shows that the principal component 1 was affected by the following variables: relative water content (RWC), electrolyte leakage (EL), photosynthetic pigments (Chl *a*, Chl *b*, Chl *t* and Car), internal CO_2_ concentration (*Ci*), CO_2_ assimilation rate, transpiration (*E*), stomatal conductance (*gs*), instantaneous carboxylation efficiency (*CEi*), number of fruits per plant (NFP), and total production per plant (TPP), with correlation coefficients greater than 0.80. On the other hand, fruit polar diameter (FPD), fruit equatorial diameter (FED), and average fruit weight (AFW) showed a significant effect for principal component 2, with correlation coefficients higher than 0.85.

**Figure 1:**
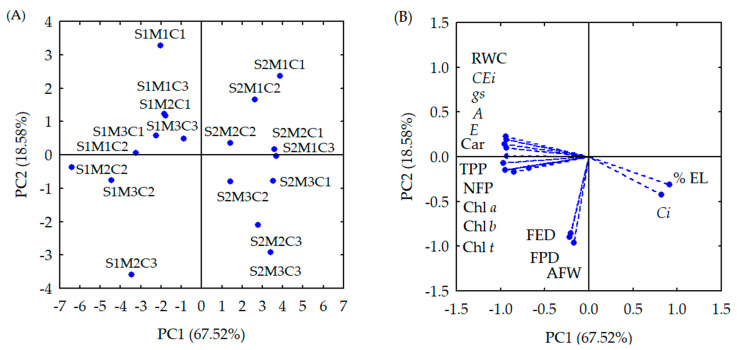
Plots of first two principal components for the three factors interactions (**A**) and analyzed variables (**B**) in sour passion fruit. S—Electrical conductivity of irrigation water (ECw), S1 (0.8 dS m^−1^); S2 (3.8 dS m^−1^); M—Application methods, M1 (Soaking); M2 (Spraying); M3 (Spraying + Soaking); C—Ascorbic acid concentration, C1 (0 mM); C2 (0.8 mM); C3 (1.6 mM); Chl *a* (Chlorophyll *a*—μg mL^−1^); Chl *b* (Chlorophyll *b*—μg mL^−1^); Chl *t* (Total chlorophyll—μg mL^−1^); Car (Carotenoids—μg mL^−1^); RWC (relative water content—%); % EL (percentage of electrolyte leakage); Ci (internal CO_2_ concentration—μmol CO_2_ m^−2^ s^−1^); A (CO_2_ assimilation rate—μmol CO_2_ m^−2^ s^−1^); E (perspiration—mmol H_2_O m^−2^ s^−1^); gs (stomatal conductance—mol H2O m^−2^ s^−1^); CEi (instantaneous carboxylation efficiency—[(μmol CO_2_ m^−2^ s^−1^) (μmol CO_2_ m^−2^ s^−1^)^−1^]. FPD (Fruit polar diameter—mm); FED (Fruit equatorial diameter—mm); AFW (Average fruit weight—g per fruit); NFP (Number of fruits per plant); TPP (Total production per plant—g per plant).

The two-dimensional projections showing the effects of treatments and variables on the first and second principal components are illustrated in Figure 1A,B. The first principal component highlights a process potentially influenced by the interaction between application methods, AsA concentrations, and ECw levels.

When analyzing PC1, it was observed that plants grown under ECw of 0.8 dS m^−1^, with AsA application by the spraying method and at concentration of 0.8 mM (S1M2C2), stood out with the highest values (Table 3) of RWC (89.51%), Chl *a* (503.33 μg mL^−1^), Chl *b* (302 μg mL^−1^), Chl *t* (805.32 μg mL^−1^), Car (105.70 μg mL^−1^), *A* (6.18 μmol CO_2_ m^−2^ s^−1^), *E* (1.27 mmol H_2_O m^−2^ s^−1^), *gs* (0.77 mol H_2_O m^−2^ s^−1^), *CEi* [(0.045 μmol CO_2_ m^−2^ s^−1^) (μmol CO_2_ m^−2^ s^−1^)^−1^], NFP (15 fruits per plant), and TPP (2011.33 g per plant).

In principal component 2 (PC2), it was observed that plants under ECw of 0.8 dS m^−1^, with AsA application by the spraying method and at concentration of 1.6 mM (S1M2C3), obtained the maximum values (Table 3) of FPD (78.95 mm), FED (69.95 mm), and AFW (159.5 g per fruit).

When comparing the results between plants subjected to the S1M2C2 and S1M2C3 treatments with those cultivated with the S1M2C1 treatment, significant increments of 54.01% (176.51 μg mL^−1^), 54.01% (105.91 μg mL^−1^), 54.01% (282.4 μg mL^−1^), 28.57% (30.2 μg mL^−1^), 3.2%, 16.82% (0.89 μmol CO_2_ m^−2^ s^−1^), 28.30% (0.28 mmol H_2_O m^−2^ s^−1^), 63.82% (0.03 mol H_2_O m^−2^ s^−1^) and 40.63% [(0.013 μmol CO_2_ m^−2^ s^−1^) (μmol CO_2_ mol^−1^)^−1^], 45.21% (4.67 fruits per plant), 57.83% (737.03 g per plant), 15.25% (10.45 mm), 27.90% (15.26 mm), and 78.21% (70.0 g per fruit) were observed for Chl *a*, Chl *b*, Chl *t*, Car, RWC, *A*, *E*, *gs*, *CEi*, NFP, TPP, FPD, FED, and AFW, respectively. In addition, there were reductions of 4.14% (11.14) in electrolyte leakage and 16.46% (27 μmol CO_2_ m^−2^ s^−1^) in internal CO_2_ concentration.

In this study, it was also observed that the increase in the electrical conductivity of irrigation water impaired the gas exchange, relative water content, contents of photosynthetic pigments, and production of sour passion fruit. When analyzing PC1, it was observed that sour passion fruit plants grown under ECw of 3.8 dS m^−1^, without application of ascorbic acid (S2M1C1), stood out with the lowest values (Table 3) of RWC (67.58%), *A* (3.18 μmol CO_2_ m^−2^ s^−1^), *E* (0.67 mmol H_2_O m^−2^ s^−1^), *gs* (0.020 mol H_2_O m^−2^ s^−1^), *CEi* (0.016 [(μmol CO_2_ m^−2^ s^−1^) (μmol CO_2_ m^−2^ s^−1^]^−1^), FPD (63.12 mm), FED (34.64 mm), and AFW (67.44 g per fruit). Lower values of Chl *a* (228.78 μg mL^−1^), Chl *b* (137.27 μg mL^−1^), Chl *t* (366.04 μg mL^−1^), Car (48.04 μg mL^−1^), and NFP (four fruits) were observed in the S2M2C1 treatment. The lowest TPP values (442.33 g per plant) were obtained in the S2M3C1 treatment.

There was a significant effect of the interaction between ascorbic acid (AsA) concentrations and water salinity (ECw) levels only on relative water content (RWC) (*p* ≤ 0.01) (Table 4). No significant effect (*p* > 0.05) of the M × AsA × ECw interaction was observed on any of the variables analyzed in sour passion fruit. In isolation, irrigation water salinity levels significantly influenced (*p* ≤ 0.01) electrolyte leakage, chlorophyll *a*, total chlorophyll, and carotenoids. AsA concentrations showed a significant effect (*p* ≤ 0.01) on Chl *a* and Chl *b* contents. Chlorophyll *b* was also significantly affected (*p* ≤ 0.01) by the application methods.

**Figure 2:**
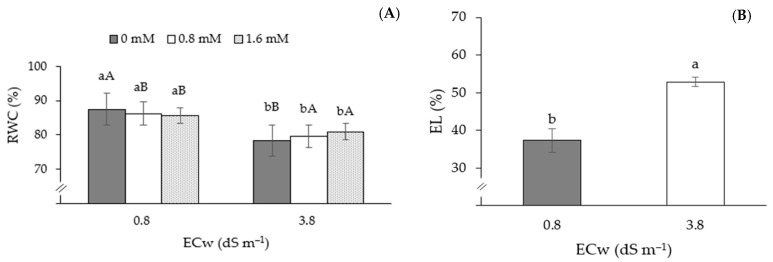
Relative water content of sour passion fruit as a function of the interaction between AsA concentrations and ECw levels (**A**), and electrolyte leakage from sour passion fruit plants as a function of irrigation water salinity (**B**), 150 days after transplanting. Different letters indicate significant differences between AsA concentrations for each electrical conductivity of irrigation water, and different uppercase letters indicate significant differences in AsA concentrations between electrical conductivities of irrigation water according to the Tukey test. (*p* ≤ 0.05). The error bars represent standard error (n = 3).

The relative water content of passion fruit plants differed significantly between water salinity levels (Figure 2A). In passion fruit plants grown under ECw of 0.8 dS m^−1^, the application of AsA at a concentration of 0 mM promoted greater RWC (87.48%) compared to that observed in plants grown under 0.8 and 1.6 mM AsA. However, in plants irrigated with ECw of 3.8 dS m^−1^, the application of AsA at concentrations of 0.8 and 1.6 mM promoted an increase in RWC in relation to plants subjected to 0 mM concentration. There was also no significant difference between the concentrations of 0.8 and 1.6 mM AsA. The salinity of the irrigation water significantly increased the electrolyte extravasation of sour passion fruit plants grown under ECw of 3.8 dS m^−1^ compared to those that received 0.8 dS m^−1^ (Figure 2B). Plants subjected to irrigation with water of 3.8 dS m^−1^ showed an increase of 15.48% compared to those that received water of 0.8 dS m^−1^.

**Figure 3:**
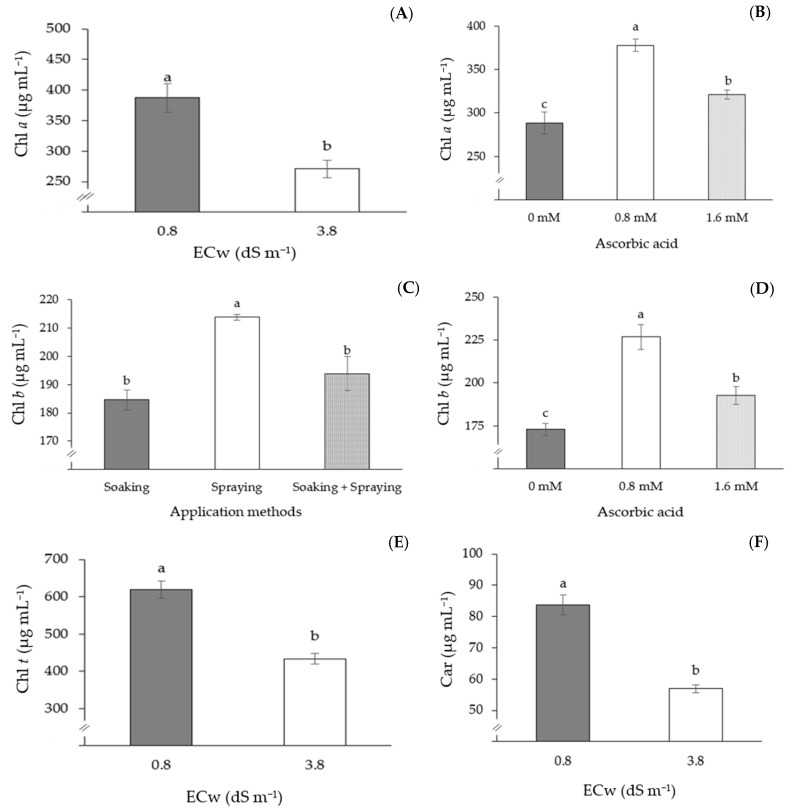
Chlorophyll *a*—Chl *a* (**A**), total chlorophyll—Chl *t* (**E**), and carotenoids—Car (**F**) depending on the electrical conductivity levels of the water (ECw); chlorophyll *a*—Chl *a* (**B**) and chlorophyll *b*—Chl *b* (**D**) depending on AsA concentrations, and Chl *b* of the sour passion fruit plant as a function of the application methods (**C**), 150 days after transplanting. Means followed by the same letter did not differ statistically from each other according to the Tukey test (*p* ≤ 0.05). The error bars represent standard error (n = 3).

Chlorophyll *a*, total chlorophyll, and carotenoid contents were affected negatively when passion fruit plants were subjected to irrigation with ECw of 3.8 dS m^−1^ (Figure 3A,E,F), with reductions of 30.0, 29.0, and 32.0% in Chl *a*, Chl *t*, and Car, respectively, compared to plants irrigated with 0.8 dS m^−1^ water. Plants that received AsA application at a concentration of 0.8 mM presented the highest values of Chl *a* (377.92 µg mL^−1^) and Chl *b* (226.75 µg mL^−1^), differing statistically from plants that received concentrations of 0 and 0.8 mM (Figure 3B,D). It was also observed that the application of AsA through spraying promoted the highest values of Chl *b* (213.86 µg mL^−1^), standing out in relation to the other application methods (Figure 3C).

The gas exchange variables of passion fruit were not significantly influenced by the interactions between AsA application methods, AsA concentrations, and irrigation water conductivity levels (Table 5). However, ECw levels significantly affected (*p* ≤ 0.01) all gas exchange variables. Internal CO_2_ concentration and stomatal conductance were also influenced (*p* ≤ 0.01) by AsA concentrations (Table 5).

**Figure 4:**
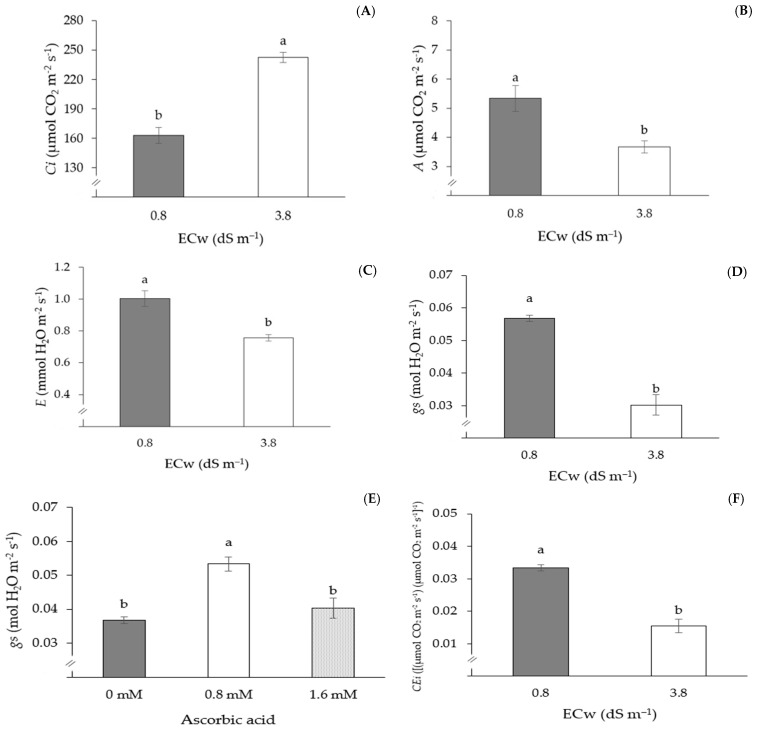
Internal CO_2_ concentration—*Ci* (**A**), CO_2_ assimilation rate—*A* (**B**), transpiration—*E* (**C**), stomatal conductance—*gs* (**D**), and instantaneous carboxylation efficiency—*CEi* (**F**) as a function of water electrical conductivity levels (ECw), and *gs* (**E**) as a function of AsA concentrations, 150 days after transplantation. Means followed by the same letter did not differ statistically from each other according to the Tukey test (*p* ≤ 0.05). The error bars represent standard error (n = 3).

The internal CO_2_ concentration increased significantly as a function of irrigation with water, with a CEa of 3.8 dS m^−1^ (Figure 4A). Plants irrigated with the highest CEa (3.8 dS m^−1^) showed an increase of 48.81% (79.56 μmol CO_2_ m^−2^ s^−1^) compared to those subjected to the lowest CEa (0.8 dS m^−1^). On the other hand, the CO_2_ assimilation rate (Figure 3B), transpiration (Figure 3C), stomatal conductance (Figure 3D), and instantaneous carboxylation efficiency (Figure 3F) were reduced by irrigation with ECw of 3.8 dS m^−1^, with reductions of 31.2% (1.66 μmol CO_2_ m^−2^ s^−1^), 24.6% (0.247 mmol H_2_O m^−2^ s^−1^), 46.8% (0.027 mol H_2_O m^−2^ s^−1^), and 53.7% (0.018 [(µmol CO_2_ m^−2^ s^−1^) (µmol CO_2_ m^−2^ s^−1^]^−1^) in *A*, *E*, *gs*, and *CEi*, respectively, when compared to plants irrigated with ECw of 0.8 dS m^−1^. It is also observed that the stomatal conductance of passion fruit plants was also influenced by AsA concentrations (Figure 3E), with an emphasis on the concentration of 0.8 mM, which promoted the highest gs value (0.053 mol H_2_O m^−2^ s^−1^), differing statistically from the other concentrations (0 and 1.6 mM).

There were significant effects from the interaction between ascorbic acid (AsA) concentrations and water salinity levels (ECw) only for on-stem diameter (SD) (*p* ≤ 0.05) (Table 6). There was no significant effect (*p* > 0.05) of the M × AsA × ECw interaction on any of the variables analyzed in sour passion fruit. On the other hand, the salinity levels of the irrigation water significantly influenced all the chlorophyll *a* fluorescence variables.

**Figure 5:**
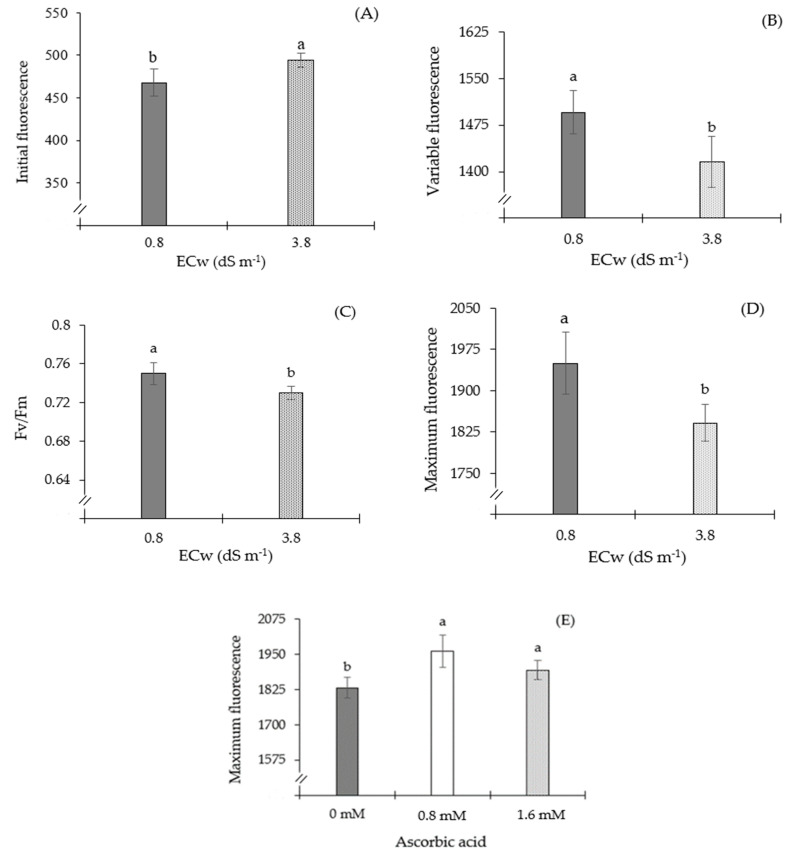
Initial fluorescence—F_0_ (**A**), variable fluorescence—Fv (**B**), quantum efficiency of photosystem II—Fv/Fm (**C**), and maximum fluorescence—Fm (**D**) of sour passion fruit cultivated under irrigation with water of different levels of electrical conductivity (ECw), and Fm as a function of ascorbic acid concentrations (**E**), at 150 days after transplanting. Means followed by the same letter did not differ statistically from each other according to the Tukey test (*p* ≤ 0.05). The error bars represent standard error (n = 3).

The salinity of irrigation water significantly increased the initial fluorescence of sour passion fruit plants grown under ECw of 3.8, compared to those that received 0.8 dS m^−1^ (Figure 2A). Plants subjected to irrigation with water of 3.8 dS m^−1^ showed a 5.64% increase in F_0_ (26.4) compared to those that received water of 0.8 dS m^−1^. Different from the result observed in the initial fluorescence (Figure 5A), sour passion fruit plants irrigated with ECw of 3.8 dS m^−1^ showed a significant reduction in their variable fluorescence compared to those irrigated with a salinity level of 0.8 dS m^−1^ (Figure 5B). When comparing the Fv of plants grown under ECw of 3.8 dS m^−1^ to that of plants subjected to water salinity of 0.8 dS m^−1^, a reduction of 5.36% was observed (80.11).

Quantum efficiency of photosystem II was also reduced by the increase in the electrical conductivity of water (Figure 5C). The Fv/Fm of plants grown under ECw of 3.8 dS m^−1^ differed significantly from those of plants that received the lowest level of water salinity (0.8 dS m^−1^). Plants irrigated with water of 3.8 dS m^−1^ had a reduction in maximum fluorescence of 5.56% (108.34) (Figure 5D) compared to those cultivated with water with the lowest salinity (0.8 dS m^−1^).

Sour passion fruit plants subjected to AsA concentrations of 0.8 and 1.6 mM obtained a higher maximum fluorescence compared to those under 0 mM of AsA (Figure 5E). When comparing the Fm of plants that received AsA concentrations of 0.8 and 1.6 mM, compared to that of plants in the control treatment (0 mM), increments of 7.10 and 3.44% were observed, respectively.

**Figure 6:**
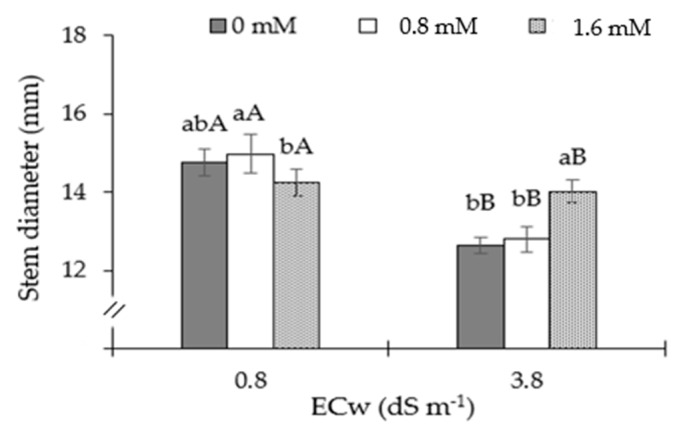
Stem diameter of sour passion fruit cultivated under irrigation with water of different levels of electrical conductivity—ECw, application methods and ascorbic acid concentrations, at 150 days after transplanting. Different letters indicate significant differences between AsA concentrations for each electrical conductivity of irrigation water, and different uppercase letters indicate significant differences in AsA concentrations between electrical conductivities of irrigation water, according to the Tukey test (*p* ≤ 0.05). The error bars represent standard error (n = 3).

The stem diameter of sour passion fruit plants differed significantly in their water salinity levels (Figure 6). In sour passion fruit plants grown under ECw of 0.8 dS m^−1^, the application of AsA at a concentration of 0.8 mM promoted a greater growth in stem diameter compared to that observed in plants grown under 1.6 mM of AsA. However, at this water salinity level, there were no significant differences in the SD of plants subjected to AsA concentrations of 0 and 1.6 mM. On the other hand, in plants irrigated with ECw of 3.8 dS m^−1^, the application of 1.6 mM of AsA resulted in greater SD growth compared to those that received concentrations of 0 and 1.8 mM.

There was no significant effect (*p* > 0.05) of the M × AsA × ECw interaction on any of the production variables analyzed in sour passion fruit (Table 7). However, a significant effect was found when the sources of variation were analyzed in isolation. The application methods significantly influenced (*p* ≤ 0.01) the polar and equatorial diameter of the fruits, the average fruit weight, and the pulp volume. AsA concentrations significantly affected (*p* ≤ 0.01) polar diameter, average fruit weight, and pulp volume. The salinity levels of irrigation water exhibited a significant effect (*p* ≤ 0.01) on the number of fruits and total production per plant. The pulp volume was also influenced by the M × AsA and AsA × ECw interactions (Table 7).

**Figure 7:**
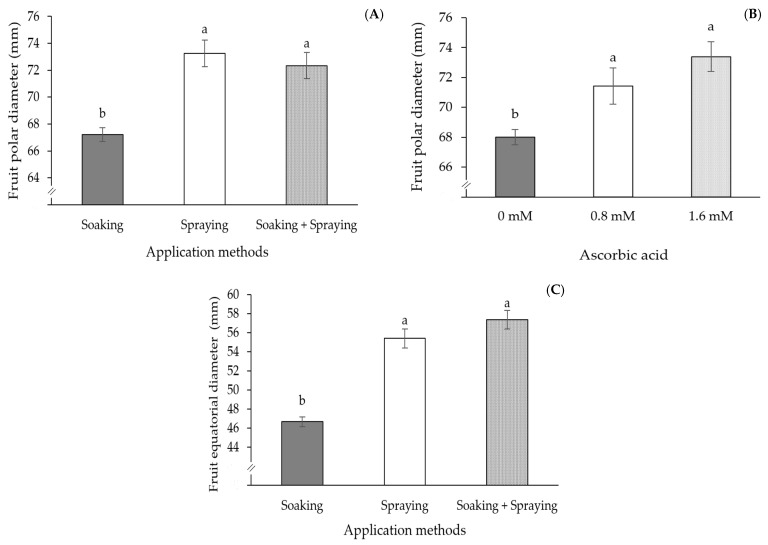
Fruit polar diameter—FPD (**A**) and fruit equatorial diameter—FED (**C**), a function of AsA application methods, and fruit polar diameter as a function of ascorbic acid concentrations (**B**), at 180 days after transplanting. Means followed by the same letter did not differ statistically from each other according to the Tukey test (*p* ≤ 0.05). The error bars represent standard error (n = 3).

The polar (Figure 7A) and equatorial diameters of passion fruit fruits (Figure 7C) of the plants subjected to the spraying, and soaking and spraying, methods differed significantly from the plants subjected to soaking alone. The highest FPD (73.26 mm) value was obtained in plants subjected to spraying and the highest FED (57.37 mm) value was recorded in plants that received AsA treatment through soaking and spraying. The FPD was also influenced by AsA concentrations (Figure 7B); it can be seen that the 0.8 and 1.6 mM concentrations did not differ from each other, but they differed significantly from the 0 mM concentration. An increase of 5.01 and 7.91% in the FPD of plants subjected to concentrations of 0.8 and 1.6 mM, respectively, was observed in relation to plants that received a concentration of 0 mM.

**Figure 8:**
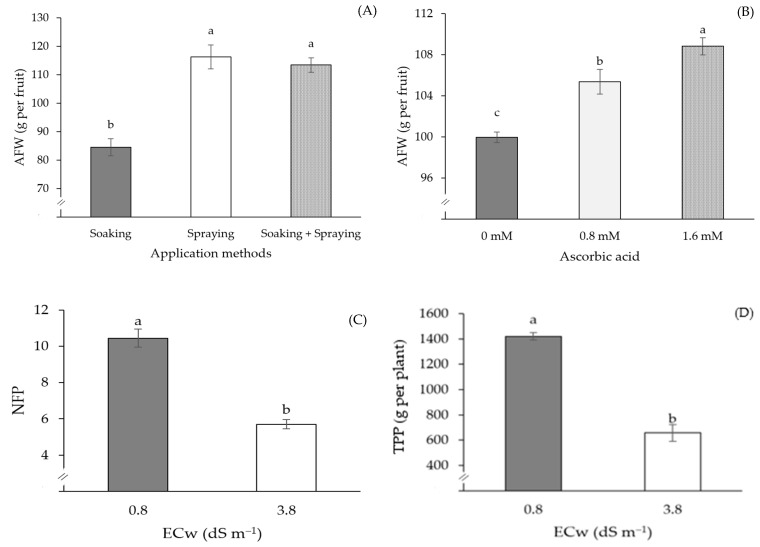
Average fruit weight—AFW as a function of the application methods (**A**) and of AsA concentrations (**B**), number of fruits per plant—NFP (**C**), and total production per plant—TPP (**D**) as a function of water electrical conductivity levels (ECw), at 180 days after transplanting. Means followed by the same letter did not differ statistically from each other according to the Tukey test (*p* ≤ 0.05). The error bars represent standard error (n = 3).

The average fruit weight of passion fruit plants subjected to the spraying, and soaking and spraying, methods did not differ from each other (Figure 8A). However, there was a significant difference in relation to the plants that were subjected to the soaking treatment, with an increase of 37.68% (31.82 g per fruit) for spraying and 34.28% (28.95 g per fruit) for soaking and spraying, in relation to plants that received only soaking. The average fruit weight was also influenced by AsA concentrations (Figure 8B). It can be seen that the plants subjected to a concentration of 1.6 mM stood out in relation to the other concentrations, presenting the highest AFW value (117.93 g), corresponding to an increase of 29.75% (27.04 g per fruit) in relation to plants submitted to a concentration of 0 mM and 12.01% (12.64 g per fruit) in relation to plants that received a concentration of 0.8 mM of AsA.

The number of fruits per plant and total production per plant were negatively affected when passion fruit plants were subjected to irrigation with ECw of 3.8 dS m^−1^ (Figure 8C,D), with reductions of 45.38% (4.74 fruits per plant) and 53.70% (762.8 g per plant), respectively, compared to plants irrigated with water of 0.8 dS m^−1^.

**Figure 9:**
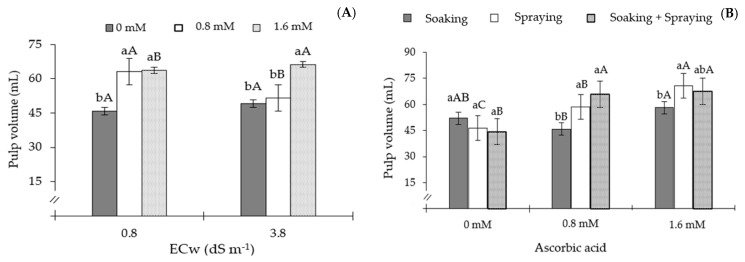
Pulp volume (PV) of passion fruit as a function of the interaction between AsA concentrations and ECw levels (**A**) and as a function of the interaction between application methods and AsA concentration (**B**), at 180 days after transplanting. Different lowercase letters indicate significant differences between AsA concentrations for each electrical conductivity of irrigation water, and different uppercase letters indicate significant differences in AsA concentrations between electrical conductivities of irrigation water, according to the Tukey test. (*p* ≤ 0.05). The error bars represent standard errors (n = 3).

Plants grown under ECw of 0.8 dS m^−1^ obtained a higher pulp volume compared to those that were subjected to water salinity of 3.8 dS m^−1^, only when they were subjected to an AsA concentration of 0.8 mM (Figure 9A). However, plants grown under AsA spraying achieved the highest pulp volume at the water salinity levels of 0.8 and 3.8 dS m^−1^, and at the AsA concentration of 1.6 mM. The increase in pulp volume in plants that received AsA at a concentration of 1.6 mM at the two water salinity levels, compared to the results of plants that did not receive AsA, was equal to 38.61% (17.72 mL) and 34.54% (17 mL), respectively. However, plants that did not receive AsA (0 mM) obtained the lowest values of pulp volume (45.89 and 49.22 mL) under water salinity of 0.8 and 3.8 dS m^−1^, respectively.

The interaction between the application methods (M) and the concentrations of ascorbic acid (Figure 9B) significantly influenced pulp volume, with the highest value (70.83 mL) obtained in plants that received AsA by the spraying method at a concentration of 1.6 mM, however, there was no significant difference in PV between plants that received AsA by spraying, and soaking and spraying. The increase in pulp volume in plants treated with AsA through the spraying, and soaking and spraying, methods was 21.95% (12.75 mL) and 16.22% (9.42 mL), respectively, compared to plants that received AsA only by soaking.

**Figure 10:**
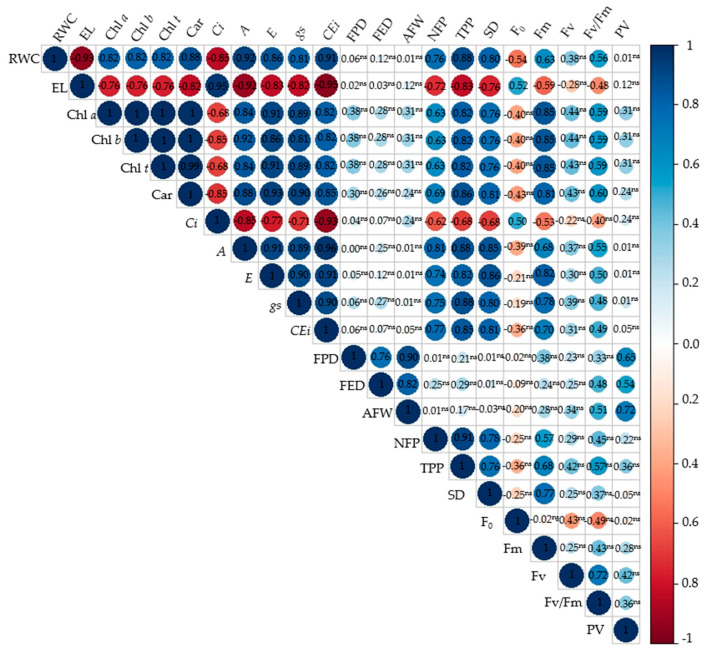
Correlation matrix obtained from the variables analyzed and the treatments applied. ns—non-significant.

The changes in morphophysiology and production for the application methods and concentrations of ascorbic acid in the mitigation of saline stress in passion fruit can be observed in the Pearson correlation matrix (Figure 10). A significant correlation was observed for most variables, with the exception of instantaneous carboxylation efficiency, polar fruit diameter, equatorial fruit diameter, average fruit weight, pulp volume, and variable fluorescence.

It was observed that the relative water content correlated positively with all variables, with the exception of electrolyte leakage (–0.93), internal carbon concentration (–0.85), and initial fluorescence (–0.54). This result is possibly linked to the respective variables being inversely proportional to the RWC. Sour passion fruit plants irrigated with ECw of 3.8 dS m^−1^ showed an increase in internal carbon concentration, which may be related to the fact that the CO_2_ absorbed in the substomatal chamber was not being properly assimilated during the photosynthetic process.

Electrolyte leakage showed negative correlations with all variables, except for internal CO_2_ concentration (0.95) and initial fluorescence (0.52). This negative correlation in most variables may be associated with the instability of cell membranes, possibly resulting in lipid peroxidation of the membrane. Photosynthetic pigments correlated positively (≥0.80) with all photosynthetic pigment variables, with the exception of internal carbon concentration, which presented a negative correlation of −0.68, −0.85, −0.68, and −0.85 for Chlorophyll *a*, chlorophyll b, total chlorophyll, and carotenoids, respectively.

A positive correlation was also observed between gas exchange parameters, the number of fruits per plant, and the total production per plant. Stomatal conductance, transpiration, the CO_2_ assimilation rate, and instantaneous carboxylation efficiency presented a high correlation of ≥0.70 in relation to the number of fruits and total fruit production. This result highlights a possible effect of ascorbic acid in mitigating saline stress in passion fruit plants. Since production is a reflection of photosynthesis, for this to occur, it directly requires efficiency in gas exchange.

## 3. Discussion

The approach with foliar application of AsA showed positive impacts on physiological activity in the current investigation, which may have improved the physiological performance and yield of passion fruit plants under stress. In comparison to other application methods, Nachtigall and Nava [18] claimed that when a product is applied by spraying, the response is nearly instantaneous and more effective in the later phases of growth, when there is a preferential absorption for fruit production compared to other application methods.

Ascorbic acid application by spraying in tomato plants exposed to salt stress increased Chl *t* (8.78%), Car (7.81%), and NFP (24%) [19]. Exogenous administration of AsA also led to an increase in RWC, indicating improved K^+^ absorption, in sorghum (*Sorghum vulgare* Pers.) and common beans (*Phaseolus vulgaris* L.) under salt stress, as reported by [20,21]. El-Sayed et al. [22] found that the foliar application of AsA also enhanced the length and diameter of olive fruits under salt stress, as well as the leaf area, total chlorophyll content in leaves, fruit and pulp percentage, and thickness.

As a result of the application of AsA at a concentration of 0.8 mM and using the spraying method, the plants under S1M2C2 treatment had the lowest internal CO_2_ concentration (137 μmol CO_2_ m^−2^ s^−1^) and the lowest electrolyte leakage (33.01%) in the leaf blades. The current investigation found that, in plants treated with AsA at a salinity level of 0.8 dS m^−1^, electrolyte leakage did not signal damage to leaf tissues.

A cell is deemed damaged when the percentage of damage (electrolyte leakage) surpasses 50%, according to Sullivan [23]. Furthermore, a faster rate of CO_2_ assimilation was discovered as a result of the decreased internal CO_2_ concentration. This happens as a result of AsA’s promotion of biomass growth and continuous plant development, which speeds up cell division and growth while strengthening membrane integrity, and lowering ion leakage and internal CO_2_ concentration. Since AsA increases the activity of many enzymes, including RuBisCO, these activities are linked to the physiological role of AsA [21,24].

The capacity of roots to absorb water and nutrients from the soil solution is limited by excess salts present in irrigation water, particularly sodium (Na^+^) and chloride (Cl^-^) ions [25,26]. This leads to osmotic stress and ionic imbalance, which in turn causes stomatal closure and reductions in transpiration, CO_2_ assimilation rate, instantaneous carboxylation efficiency, and water use efficiency [3,27]. Other investigations involving various fruit crops, including mandarin [28], West Indian cherry [29], soursop [30], guava [31], and cashew [32], have also noted the decrease in gas exchange as measured by gs, E, and A, with an increase in the electrical conductivity of irrigation water. However, sour passion fruit plants’ decreased ability to synthesize photosynthetic pigments as a result of salt stress was also observed by [33].

In research assessing the effects of water salinity on the sour passion fruit crop, the ‘Guinezinho’ accession, Andrade et al. [34] found that an increase in ECw from 0.7 dS m^−1^ resulted in a lower number of fruits and a lower mean fruit weight. In a different investigation, Lima et al. [33] discovered that sour passion fruit under salt stress had smaller polar and equatorial diameters. According to Souza et al. [35], electrical conductivity of 0.3 dS m^−1^ decreased the sour passion fruit plants’ RWC, chlorophyll *a* and *b* levels, CO_2_ assimilation rate, and instantaneous water use efficiency.

The increase in F0 in the present study (Figure 5A) indicates that salt stress may have damaged the photosynthesis system, leading to a reduction in light energy. This condition is indicative of a state of oxidation of quinone (the major electron receptor) in the reaction center (P680), which prevents photosystem II from transferring energy [29].

Conversely, Fv (Figure 5B) was decreased through irrigation using 3.8 dS m^−1^ ECw. This decrease indicates a restriction in the activation of the electron transport chain, which plays a crucial role in the synthesis of energy in the form of ATP and NADPH for the Calvin cycle. This reduction is related to the active potential energy in the photosystem. As a result, a plant’s ability to photosynthesize is lowered [36,37]. In research with soursop (*Annona muricata* L.) plants irrigated with salty water (ECw ranging from 0.8 to 4.0 dS m^−1^), Silva et al. [30] discovered that, at 480 days after transplanting, there was a drop in variable fluorescence of 5.21% per unit increase of ECw, starting at 0.8 dS m^−1^.

The ratio Fv/Fm showed a similar impact to that seen in Fv (Figure 5C), i.e., a decrease promoted by irrigation with water that had a higher salinity (3.8 dS m^−1^). This scenario shows that sour passion fruit plants can sustain photochemical damage. A portion of the light energy in the thylakoid membrane, linked to the metabolic consequences of salt stress, increases the generation of reactive oxygen species (ROS), which in turn degrades photosynthetic pigments in the reaction center [38,39]. When precocious dwarf cashew seedlings were irrigated with saline waters (ECw varying from 0.4 to 3.6 dS m^−1^), Silva et al. [32] observed a 19.05% decrease in photosystem II quantum efficiency in the cashew plants.

The results of the present study also revealed that passion fruit plants that were irrigated with ECw of 3.8 dS m^−1^ had less maximum fluorescence (Figure 5D). Salt stress most likely limited the amount of energy absorbed in the light reaction centers because it causes an imbalance in the plant’s metabolic activities by causing an excessive build-up of harmful ions. Reactive oxygen species are produced as a result, which may restrict energy efficiency [39]. In a study conducted by Silva et al. [31] using soursop (*Annona muricata* L.) cv. Morada Nova plants irrigated with salty water (ECw from 0.8 to 4.0 dS m^−1^), it was discovered that an increase in ECw levels caused a reduction of 10.9% per unit increment of electrical conductivity in maximum fluorescence.

Conversely, applying AsA at 0.8 and 1.6 mM concentrations promoted an increase in Fm (Figure 5E). Ascorbic acid is responsible for the rise in maximum fluorescence because it acts as a cofactor of violaxanthin de-epoxidase, an enzyme that is essential in the xanthophyll cycle. This cycle represents a mechanism for dissipating excessive light energy that could cause damage to chloroplasts. In the xanthophyll cycle, there is a reversible conversion of violaxanthin to antheraxanthin and zeaxanthin, pigments with the ability to dissipate excess energy in the form of heat, i.e., AsA plays a crucial role in assisting plants in regulating the flow of energy in chloroplasts, thus preventing photoinhibition [40].

Excessive ion accumulation lowers the free energy potential of water in the soil, which makes it more difficult for plants to absorb nutrients and water. Genes that are responsible for the changeover in the synthesis of lignin, suberin, and cell wall polysaccharides may change as a result of this circumstance. These modifications negatively affect the rates of elongation and cell division in tissues, and consequently growth [41,42]. This effect may have contributed to the reduction in the stem diameter of passion fruit plants (Figure 6).

Restrictions in water absorption and the harmful effects of ions (Na^+^ and Cl^−^), which accumulate in stem tissues during plant development and might change osmotic and ionic balance, may also be linked to the inhibition of plant growth [43,44]. The restrictions in the rate of CO_2_ assimilation of plants irrigated with water having an electrical conductivity of 3.8 dS m^−1^, which modifies the partitioning of photoassimilates, are the cause of the inhibition in the growth of sour passion fruit plants. Furthermore, one factor that leads to growth limitation in plants grown under stress is the energy required to sustain metabolic activities. In a study carried out by Lima et al. [45] with passion fruit irrigated with saline water (ECw of 0 and 150 mM), the authors concluded that the higher salinity level of irrigation water resulted in the inhibition of the growth of the stem diameter.

The pulp volume of passion fruit fruits was likewise observed to be decreased by irrigation with an ECw of 3.8 dS m^−1^ in the current study (Figure 9A). As a protective measure against excessive water loss, plants partially close their stomata due to an increase in the concentration of salts in the soil. This restricts the entry of CO_2_ into the substomatal chamber, which in turn affects carboxylation efficiency and the efficient use of water [46]. Ramos et al. [47], in a study with the sour passion fruit cv. BRS Rubi do Cerrado under irrigation with saline water (ECw from 0.6 to 3.0 dS m^−1^), observed a decrease of 49.84% in the pulp yield of fruits of plants subjected to the higher ECw.

Despite the reduction in pulp volume due to the salinity of the irrigation water (Figure 9A), it was observed that spraying AsA at a concentration of 1.6 mM was able to promote an increase in pulp volume (Figure 9B). This increase in pulp volume may be associated with the role of AsA as an antioxidant, since AsA has the ability to react directly with several reactive oxygen species, resulting in an improvement of the physiological functions of plants [48]. In addition, the application of AsA by spraying induces a practically instantaneous and more efficient response during the advanced stages of growth [18].

In general, the results obtained in this study point to the potential benefits of AsA at concentrations of 0.8 and 1.6 mM, in conjunction with the foliar spraying method, as a means of reducing the negative effects of salt stress on sour passion fruit BRS GA1 plants irrigated with moderately saline waters (ECw of 0.8 and 3.8 dS m^−1^).

## 4. Materials and Methods

### 4.1. Experimental Site

**Figure 11:**
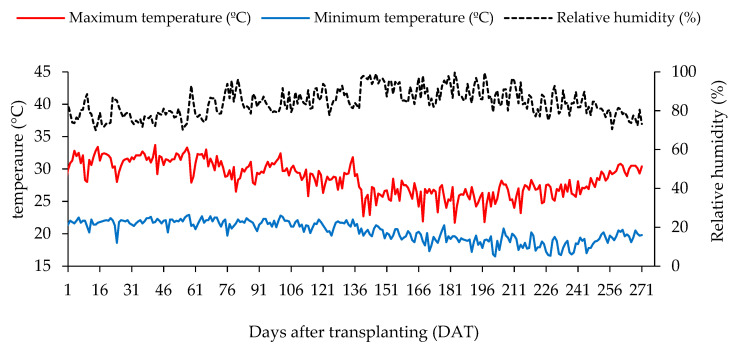
Maximum and minimum temperatures, and average relative humidity, observed in the indoor area of the greenhouse during the experimental period (5 January to 3 October 2022).

The experiment was conducted from January to October 2022 in a controlled environment at the Academic Unit of Agricultural Engineering, Federal University of Campina Grande, in Campina Grande, Paraíba, Brazil (7°15′18″ S, 35°52′28″ W, at an average altitude of 550 m). The climate in Campina Grande is classified as type As’, characterized by warm and humid conditions with rain in winter and autumn. Figure 11 presents the maximum and minimum air temperatures, as well as the average relative humidity recorded during the study.

### 4.2. Experimental Design and Treatments

The experimental design was randomized blocks, arranged in a 3 × 3 × 2 factorial scheme, corresponding to three methods of application of ascorbic acid—M (soaking, spraying, and soaking and spraying), three concentrations of ascorbic acid—AsA (0, 0.8, and 1.6 mM) and two electrical conductivities of the irrigation water—ECw (0.8 and 3.8 dS m^−1^) (Table 8), with three replicates.

Ascorbic acid (AsA) concentrations of 0.8 and 1.6 mM were based on a study conducted by Fatah and Sadek [16], adjusted to millimolar (mM).

### 4.3. Plant Material and Seedling Formation

The cultivar BRS GA1 was used in this study. The fruit exhibits a yellow color and has an oblong shape, with a slightly flattened base and apex, and an average weight ranging from 120 to 350 g, while the pulp yield is approximately 40% [49]. Sour passion fruit seedlings were produced in plastic bags with dimensions of 10 × 20 cm using 2.6 kg of substrate consisting of 2.18 kg of soil (84%), 0.39 kg of sand (15%), and 0.03 kg of humus (1%). Prior to sowing, sour passion fruit seeds were soaked in ascorbic acid solutions (0, 0.8, and 1.6 mM) for a period of 24 h in the dark.

Ascorbic acid concentrations were prepared from the dissolution of ascorbic acid in distilled water and stored in a dark environment [50]. Subsequently, three seeds were sown in each plastic bag at 3 cm depth, spaced equidistantly. After 40 days of sowing, thinning was carried out, keeping only the most vigorous plant in each bag. Before sowing, the soil moisture content was brought to field capacity using water according to the treatment.

Irrigation was carried out daily, applying a volume of water to each bag to keep the substrate moisture close to field capacity. The volume applied was determined based on the water needs of the plants, calculated by the water balance, considering the volume drained from the previous irrigation. Every 20 days, the volume to be applied was incremented by a leaching fraction of 0.10 to avoid excessive accumulation of salts in the root zone [51].

### 4.4. Experiment Setup and Conduction

From the moment the plants began to produce tendrils (at 70 days after sowing—DAS), they were transplanted into pots adapted as drainage lysimeters, with a capacity of 120 L. The lysimeters were perforated at the base to allow drainage, and connected to a 16-mm-diameter transparent drain (green color). The tip of the drain inside the lysimeter was wrapped with a non-woven geotextile (Bidim OP 30, Geomembrana^®^, Guarulhos—SP, Brazil) to prevent clogging by soil material.

A plastic bottle was placed below each drain to collect drained water and determine water consumption by the plant. The lysimeters were filled with a 1.0 kg layer of crushed stone followed by 160 kg of *Neossolo Regolítico* (Psamment) with sandy loam texture from the municipality of Lagoa Seca, PB, Brazil (7°10′8″ South latitude, 35°51′20″ West longitude), collected at 0–30 cm depth. Before starting the experiment, chemical and physical–hydraulic attributes of the soil were determined according to the methodology proposed by Teixeira et al. [52]: pH (H_2_O) = 4.93; organic matter = 9.3 g dm^−3^; P = 10.7 g dm^−3^; K^+^ = 0.23 cmol_c_ kg^−1^; Na^+^ = 0.51 cmol_c_ kg^−1^; Ca^2+^ = 1.77 cmol_c_ kg^−1^; Mg^2+^ = 1.60 cmol_c_ kg^−1^; Al^3+^ = 2.64 cmol_c_ kg^−1^; H^+^ = 0.51 cmol_c_ kg^−1^. ECse = 1.15 dS m^−1^; CEC = 7.23 cmol_c_ kg^−1^; SAR = 0.38 mmol L^−1^; ESP = 7.05%; particle-size fraction: sand = 760.9 g kg^−1^; silt = 164.5 g kg^−1^; clay = 74.6 g kg^−1^; moisture at 33.42 KPa = 13.07 dag kg^−1^; and moisture at 1519.5 KPa = 5.26 dag kg^−1^.

### 4.5. Preparation of Waters and Irrigation Management

Saline solutions were prepared by adding NaCl, CaCl_2_·2H_2_O, and MgSO_4_·7H_2_O salts to the local water supply in Campina Grande (ECw = 0.38 dS m^−1^), maintaining a ratio of 7:2:1, which reflects the typical composition of water in the north-eastern semi-arid region [53]. Irrigation with these saline waters started 33 days after transplanting (DAT), with applications every 3 days. Each lysimeter received water according to the specific treatments, aiming to keep soil moisture near field capacity. The volume of water applied was based on the plants’ water needs, as determined by the water balance.

### 4.6. Preparation and Application of Ascorbic Acid Solutions

Only distilled water was used to represent the concentration of 0 mM. Prior to sowing, the sour passion fruit seeds corresponding to the soaking, and soaking and spraying, treatments were soaked in ascorbic acid solutions (0, 0.8, and 1.6 mM) for a period of 24 h in the dark. This period was determined by a test conducted before the experiment, with testing times of 8, 12, and 24 h of soaking, and by evaluating the characteristics of germination and seedling quality.

Foliar applications of AsA began at 30 DAT and were later performed at 30-day intervals, totaling 4 applications, carried out with a Jacto XP-12 knapsack sprayer (Jacto^®^, Pompeia—SP, Brazil) and using a pump with working pressure (maximum) of 6 bar, with a JD-12 nozzle (Jacto^®^, Pompeia—SP, Brazil) and flow rate of approximately 770 mL min^−1^. Spraying with AsA was performed on the abaxial and adaxial sides of the leaves between 5:00 p.m. and 5:45 p.m. until the plants were fully wetted. The average volume of spray applied during the experiment was 583 mL per plant.

### 4.7. Formative Pruning Management

The rows and plants were spaced by 1.5 m using a vertical trellis system. Formative pruning commenced 15 days after transplanting, removing all lateral shoots and leaving only the main stem.

**Figure 12:**
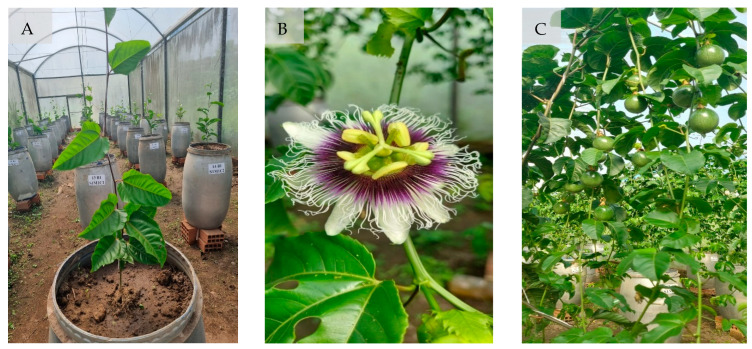
Arrangement of sour passion fruit plants in the experimental area in the different phenological stages: vegetative stage (**A**), flowering stage (**B**) and fruiting stage (**C**).

When the plants reached a height of 10 cm above the trellis (2.20 m), the apical bud was pruned to encourage two secondary branches, which were then trained in opposite directions until they grew to 0.75 m. Once this length was achieved, the apical buds of the secondary branches were pruned to promote the growth of tertiary branches, which were trained down to 30 cm from the ground, forming a curtain for inflorescence development. Throughout the experiment, tendrils and unwanted branches were removed to ensure optimal crop development (Figure 12).

### 4.8. Fertilization Management

Fertilization with nitrogen, phosphorus, and potassium was based on the recommendation of Cavalcante et al. [54] for the sour passion fruit crop, using 160 g of N, 140 g of P_2_O_5_, and 480 g of K_2_O per plant per year. N and K were applied as top-dressing divided into 18 equal portions and applied at 15-day intervals; P was applied at once, as basal. Urea, single superphosphate, and potassium chloride were used as sources of N, P, and K, respectively.

Micronutrient fertilization was carried out by foliar sprays with a solution containing 1 g L^−1^ of Dripsol^®^ [Mg (1.1%); Zn (4.2%); B (0.85%); Fe (3.4%); Mn (3.2%); Cu (0.5%); Mo (0.05%)] at 30, 60, 90, 120, and 150 DAT.

### 4.9. Variables Analyzed

#### 4.9.1. Physiological Variables

At 150 days after transplanting (DAT), treatment effects were evaluated through relative water content, electrolyte leakage, gas exchange, chlorophyll *a* fluorescence, photosynthetic pigments, and growth in stem diameter.

Relative water content (RWC) was determined according to the methodology of Weatherley [55]. Electrolyte leakage from the leaf blades was determined according to Scotti-Campos et al. [56].

##### Gas Exchange

Gas exchange was determined by stomatal conductance—*gs* (mol H_2_O m^−2^ s^−1^), transpiration—*E* (mmol H_2_O m^−2^ s^−1^), internal CO_2_ concentration—*Ci* (μmol CO_2_ m^−2^ s^−1^), CO_2_ assimilation rate—*A* (μmol CO_2_ m^−2^ s^−1^), instantaneous water use efficiency (WUEi) (*A*/*E*) [(μmol CO_2_ m^−2^ s^−1^) (mmol H_2_O m^−2^ s^−1^)^−1^], and instantaneous carboxylation efficiency—*CEi* (*A*/*Ci*) ([(μmol CO_2_ m^−2^ s^−1^) (μmol CO_2_ mol^−1^)^−1^]), between 06:00 and 09:00 a.m. on fully expanded leaves located in the upper third, using a portable infrared carbon dioxide analyzer (IRGA), model LCPro+ Portable Photosynthesis System^®^ (ADC BioScientific Limited, Hoddesdon, United Kingdom), with temperature control at 25 °C, irradiation of 1200 μmol photons m^−2^ s^−1^, and airflow of 200 mL min^−1^.

##### Chlorophyll *a* Fluorescence

Chlorophyll *a* fluorescence was measured by initial fluorescence (F_0_), maximum fluorescence (Fm), variable fluorescence (Fv), and quantum efficiency of photosystem II (Fv/Fm), determined by a pulse-modulated fluorometer, model OS5p from Opti Science (Hudson, NH, USA), between 06:00 and 09:00 a.m. on fully expanded leaves located in the upper third. Leaf clips were placed on these leaves and, after a period of 30 min of adaptation to the dark, chlorophyll *a* fluorescence was determined.

##### Photosynthetic Pigments

Photosynthetic pigments were quantified according to Arnon [57], using extracts obtained from disc samples of the third mature leaf at the apex of the plant. These extracts were used to quantify the contents of chlorophyll *a*, chlorophyll *b*, total chlorophyll, and carotenoids in the solutions with a spectrophotometer at absorbance wavelengths (ABS) of 470, 646, and 663 nm.

#### 4.9.2. Growth Variable

The stem diameter (SD) of sour passion fruit plants was measured close to the plant collar (5.0 cm from the ground) with a digital caliper.

#### 4.9.3. Production Variable

Fruit harvesting began at 150 days after transplanting, and was completed at 180 DAT. The fruits were collected by individually removing the plant, cutting the peduncle when the fruit color changed from green to partially yellow, and before detaching from the mother plant [58,59]. Even when they were not yet fully ripe, the fruits of each plant were individually identified with the numbers corresponding to the pots. Harvests were carried out daily in the morning, followed by the transport of the fruits to the Irrigation and Drainage Laboratory (LEID) for physical characterization.

Sour passion fruit production was evaluated by determining the number of fruits per plant (NFP), obtained by directly counting the fruits that reached the full maturity stage. The average fruit weight (AFW) was obtained by the ratio between the production per plant, the total number of fruits, and the total production per plant (TPP) using a benchtop scale, fruit polar diameter (FPD), fruit equatorial diameter (FED), and digital caliper, and measuring as the fruits were harvested, with the results expressed in millimeters (mm). Pulp volume (PV) was also measured, using a 250 mL beaker.

### 4.10. Statistical Analysis

The multivariate structure of the results was analyzed using the principal component analysis technique. This analysis synthesizes the relevant information contained in the original dataset into fewer dimensions. The new dimensions are generated from linear combinations of the original variables, based on the eigenvalues (λ ≥ 1.0) found in the correlation matrix. These dimensions explain a significant percentage greater than 10% of the total variance of the data [60].

Once the dimensions were reduced, the original data of the variables of each component were subjected to a multivariate analysis of variance (MANOVA) using the Hotelling test [61] at 0.05 probability level. This was done for the factors’ electrical conductivity of irrigation water, ascorbic acid concentrations, and ascorbic acid application methods, and to check the interaction between these factors. Only the variables that showed a correlation coefficient greater than or equal to 0.6 were maintained in each principal component (PC) [62]. Variables that did not show a correlation coefficient greater than or equal to 0.6 were subjected to analysis of variance through the F test at 0.05 and 0.01 probability levels and, in cases of significance, the means were compared using the Tukey test at 0.05 probability level, and using the statistical software SISVAR v.5.6 [63].

## 5. Conclusions

Foliar spraying of ascorbic acid at a concentration of 0.8 mM mitigates the effects of salt stress on electrolyte leakage, relative water content in leaves, photosynthetic pigments, gas exchange, and the total fruit production of sour passion fruit at 150 days after transplanting. An increase in the electrical conductivity of water above 3.8 dS m^−1^ negatively affects chlorophyll *a* fluorescence in plants that did not receive ascorbic acid treatment. Sour passion fruit plants grown with water of 0.8 dS m^−1^ and foliar application of 0.8 mM of ascorbic acid achieved the highest pulp volume and the highest growth in stem diameter.

The results obtained in this study confirm the hypothesis that ascorbic acid, when applied in the appropriate concentration and method, can act as a signaling molecule and mitigate the effect of the salinity of the irrigation water on the sour passion fruit. However, further studies are needed to analyze the effects of water salinity on the post-harvest quality of the fruits and to understand how ascorbic acid acts in the signaling of salt stress through enzymatic analyses, in addition to validating the results in field research.

## Figures and Tables

**Table 1:** Eigenvalues, the percentage of total variance explained in the multivariate analysis of variance, and the correlation coefficients (r) between the original variables and the principal components.

	Principal Components (PCs)	
	PC1	PC2
Eigenvalues (λ)	10.80	2.97
Percentage of total variance (S^2^%)	67.52	18.58
Hotelling test (T^2^) for interaction (ECw × M)	0.01	0.01
Hotelling test (T^2^) for interaction (ECw × C)	0.01	0.01
Hotelling test (T^2^) for interaction (M × C)	0.01	0.01
Hotelling test (T^2^) for interaction (ECw × M × C)	0.03	0.05

**Table 2:** Correlation coefficients (r) between the original variables and the principal components.

PCs	Correlation Coefficients (r)							
	RWC	EL	Chl *a*	Chl *b*	Chl *t*	Car	*Ci*	*A*
PC1	−0.929 *	0.909 *	−0.939 *	−0.937 *	−0.938 *	−0.966 *	0.819 *	−0.947 *
PC2	0.187	−0.308	−0.146	−0.147	−0.149	−0.068	0.417	0.139
**PCs**	**Correlation Coefficients (r)**							
	***E***	***gs***	***CEi***	**FPD**	**FED**	**AFW**	**NFP**	**TPP**
PC1	−0.928 *	−0.929 *	−0.937 *	0.219	−0.209	−0.169	−0.809 *	−0.849 *
PC2	0.098	0.078	0.219	−0.898 *	−0.859 *	−0.958 *	−0.119	−0.169

PC1—Principal component 1; PC2—Principal Component 2. *—Variable with Pearson’s correlation coefficient (r ≥ 0.80) considered for PC. Correlation coefficient determined considering the three repetitions (n = 3).

**Table 3:** Mean values of variables under the effect of studied treatments.

Treatments	Variables							
	RWC	EL	Chl *a*	Chl *b*	Chl *t*	Car	*Ci*	*A*
S1M1C1	86.48 ± 0.5 a	36.11 ± 1.8 a	341.11 ± 11.8 a	204.67 ± 11.8 a	545.77 ± 9.8 a	78.80 ± 1.8 a	160 ± 1.8 a	5.12 ± 0.9 a
S1M2C1	86.31 ± 0.9 a	37.15 ± 1.5 a	326.82 ± 13.2 a	196.09 ± 5.8 a	522.92 ± 8.7 a	75.50 ± 1.6 a	164 ± 2.9 a	5.29 ± 1.3 a
S1M3C1	83.45 ± 1.1 a	35.94 ± 1.8 a	349.25 ± 12.5 a	209.55 ± 9.2 a	558.80 ± 6.8 a	80.68 ± 2.4 a	173 ± 3.1 a	5.32 ± 1.1 a
S1M1C2	79.66 ± 1.1 a	35.15 ± 1.6 a	402.66 ± 22.6 a	241.60 ± 12.8 a	644.26 ± 7.9 a	84.56 ± 3.1 a	144 ± 4.2 a	5.21 ± 0.9 a
S1M2C2	89.51 ± 2.2 a	33.01 ± 2.1 a	503.33 ± 30.1 a	302.00 ± 14.8 a	805.32 ± 9.8 a	105.70 ± 2.8 a	137 ± 4.9 a	6.18 ± 1.2 a
S1M3C2	83.89 ± 1.4 a	35.54 ± 1.2 a	427.83 ± 26.3 a	256.70 ± 19.5 a	684.53 ± 7.8 a	89.84 ± 3.9 a	146 ± 3.8 a	5.87 ± 1.1 a
S1M1C3	83.50 ± 1.4 a	40.05 ± 5.1 a	342.26 ± 22.1 a	205.36 ± 22.8 a	547.62 ± 9.2 a	71.88 ± 4.1 a	156 ± 7.1 a	5.35 ± 0.8 a
S1M2C3	83.28 ± 0.9 a	41.36 ± 4.3 a	427.83 ± 14.4 a	256.70 ± 29.9 a	684.53 ± 8.6 a	89.84 ± 5.7 a	191 ± 6.4 a	5.01 ± 1.5 a
S1M3C3	82.69 ± 0.8 a	41.72 ± 4.3 a	363.65 ± 13.7 a	218.19 ± 14.7 a	581.85 ± 6.5 a	76.37 ± 3.2 a	196 ± 5.7 a	4.66 ± 0.8 a
S2M1C1	67.58 ± 2.6 a	48.82 ± 6.8 a	238.78 ± 14.7 a	143.27 ± 19.7 a	382.04 ± 6.5 a	50.14 ± 4.5 a	198 ± 4.9 a	3.18 ± 1.2 a
S2M2C1	70.77 ± 1.3 a	50.90 ± 4.2 a	228.78 ± 21.0 a	137.27 ± 14.4 a	366.04 ± 7.1 a	48.04 ± 3.8 a	210 ± 4.3 a	3.68 ± 1.3 a
S2M3C1	68.43 ± 1.1 a	51.11 ± 3.2 a	244.47 ± 19.7 a	146.68 ± 10.2 a	391.16 ± 8.6 a	51.34 ± 4.2 a	227 ± 3.5 a	3.60 ± 1.2 a
S2M1C2	69.54 ± 1.7 a	51.43 ± 1.8 a	281.86 ± 13.7 a	169.12 ± 21.9 a	450.98 ± 8.7 a	59.19 ± 3.1 a	228 ± 9.8 a	4.29 ± 0.9 a
S2M2C2	73.40 ± 1.2 a	52.55 ± 1.7 a	352.33 ± 14.4 a	211.40 ± 24.5 a	563.73 ± 7.6 a	73.99 ± 8.6 a	233 ± 9.7 a	3.69 ± 0.8 a
S2M3C2	69.81 ± 2.2 a	52.98 ± 1.5 a	299.48 ± 16.8 a	179.69 ± 19.6 a	479.17 ± 4.9 a	62.89 ± 9.1 a	257 ± 8.7 a	4.43 ± 0.7 a
S2M1C3	68.47 ± 2.7 a	53.36 ± 1.7 a	239.58 ± 17.9 a	143.75 ± 14.5 a	383.33 ± 3.9 a	50.31 ± 2.4 a	259 ± 6.5 a	3.55 ± 0.7 a
S2M2C3	68.29 ± 2.1 a	55.67 ± 1.6 a	299.48 ± 18.5 a	179.69 ± 17.9 a	479.17 ± 7.8 a	62.89 ± 3.9 a	283 ± 5.4 a	3.41 ± 0.9 a
S2M3C3	67.81 ± 1.8 a	58.56 ± 2.1 a	254.56 ± 16.8 a	152.73 ± 17.1 a	407.29 ± 9.8 a	53.46 ± 8.1 a	288 ± 6.1 a	3.21 ± 0.5 a
**Treatments**	**Variables**							
	***E***	***gs***	***CEi***	**FPD**	**FED**	**AFW**	**NFP**	**TPP**
S1M1C1	0.96 ± 0.3 a	0.057 ± 0.02 a	0.032 ± 0.01 a	63.97 ± 1.7 a	41.31 ± 2.4 a	67.25 ± 3.1 a	11.33 ± 0.7 a	1500.0 ± 32.2 a
S1M2C1	0.99 ± 0.2 a	0.047 ± 0.06 a	0.032 ± 0.01 a	68.50 ± 1.8 a	54.69 ± 3.4 a	89.50 ± 3.4 a	10.33 ±0.9 a	1274.3 ± 54.1 a
S1M3C1	0.96 ± 0.3 a	0.053 ± 0.04 a	0.031 ± 0.02 a	67.96 ± 1.5 a	56.82 ± 2.3 a	102.17 ± 3.4 a	12.00 ± 1.2 a	1396.7 ± 38.9 a
S1M1C2	1.02 ± 0.2 a	0.070 ± 0.02 a	0.036 ± 0.03 a	72.53 ± 1.1 a	58.58 ± 2.2 a	102.33 ± 2.9 a	8.67 ± 0.9 a	1272.2 ± 41.4 a
S1M2C2	1.27 ± 0.1 a	0.077 ± 0.01 a	0.045 ± 0.02 a	75.90 ± 1.1 a	52.36 ± 3.1 a	120.13 ± 5.1 a	15.00 ± 1.1a	2011.3 ± 81.9 a
S1M3C2	1.13 ± 0.1 a	0.067 ± 0.02 a	0.040 ± 0.01 a	74.74 ± 1.4 a	62.26 ± 4.9 a	118.33 ± 3.4a	9.67 ± 0.5 a	1418.3 ± 56.4 a
S1M1C3	0.93 ± 0.3 a	0.050 ± 0.02 a	0.034 ± 0.02 a	67.57 ± 2.3 a	55.97 ± 3.7 a	84.17 ± 2.8 a	11.67 ± 0.3 a	1284.0 ± 46.1 a
S1M2C3	0.88 ± 0.4 a	0.047 ± 0.03 a	0.026 ± 0.01 a	78.95 ± 1.2 a	69.95 ± 2.7 a	159.50 ± 3.2 a	8.33 ± 0.4 a	1409.0 ± 57.9 a
S1M3C3	0.89 ± 0.3 a	0.043 ± 0.02 a	0.024 ± 0.01 a	72.24 ± 1.8 a	43.23 ± 3.7 a	102.00 ± 4.1a	7.00 ± 0.8 a	1218.0 ± 68.7 a
S2M1C1	0.67 ± 0.5 a	0.020 ± 0.02 a	0.016 ± 0.02 a	63.12 ± 2.1 a	34.64 ± 4.8 a	67.44 ± 2.7 a	5.67 ± 0.2 a	567.7 ± 29.8 a
S2M2C1	0.72 ± 0.3 a	0.023 ± 0.03 a	0.018 ± 0.01 a	72.18 ± 1.9 a	49.77 ± 3.4 a	105.00 ± 3.1 a	4.00 ± 0.4 a	475.3 ± 29.5 a
S2M3C1	0.72 ± 0.4 a	0.021 ± 0.02 a	0.016 ± 0.01 a	72.32 ± 1.6 a	59.15 ± 2.2 a	114.00 ± 2.9a	4.67 ± 0.6 a	442.3 ± 31.8 a
S2M1C2	0.77 ± 0.2 a	0.037 ± 0.02 a	0.019 ± 0.02 a	68.07 ± 2.1 a	39.18 ± 3.1 a	76.50 ± 4.2 a	4.00 ± 0.7 a	456.0 ± 41.2 a
S2M2C2	0.95 ± 0.8 a	0.032 ± 0.01 a	0.016 ± 0.01 a	67.95 ± 1.8 a	45.55 ± 2.4 a	104.50 ± 3.7 a	4.00 ± 0.8 a	477.3 ± 35.6 a
S2M3C2	0.85 ± 0.6 a	0.037 ± 0.02 a	0.017 ± 0.02 a	69.30 ± 2.2 a	58.05 ± 1.8 a	110.00 ± 2.8 a	11.00 ± 1.2 a	1085.3 ± 58.9 a
S2M1C3	0.70 ± 0.2 a	0.027 ± 0.01 a	0.014 ± 0.01 a	68.06 ± 3.4 a	50.36 ± 3.2 a	109.00 ± 3.1 a	6.00 ± 0.9 a	824.0 ± 54.6 a
S2M2C3	0.72 ± 0.1 a	0.040 ± 0.02 a	0.012 ± 0.01 a	76.05 ± 2.1 a	60.07 ± 1.4 a	119.00 ± 2.4 a	4.00 ± 1.1 a	745.0 ± 41.6 a
S2M3C3	0.71 ± 0.3 a	0.035 ± 0.03 a	0.011 ± 0.01 a	77.49 ± 2.1 a	64.70 ± 1.9 a	133.88 ± 5.8 a	8.00 ± 1.4 a	845.7 ± 31.4 a

S—Electrical conductivity of irrigation water (ECw), S1 (0.8 dS m^−1^); S2 (3.8 dS m^−1^); M—Application methods, M1 (Soaking); M2 (spraying); M3 (Spraying + Soaking); C—Ascorbic acid concentration, C1 (0 mM); C2 (0.8 mM); C3 (1.6 mM); RWC (relative water content—%); EL (percentage of electrolyte leakage); Chl *a* (Chlorophyll *a*—μg mL^−1^); Chl *b* (Chlorophyll *b*—μg mL^−1^); Chl *t* (Chlorophyll total—μg mL^−1^); Car (Carotenoids—μg mL^−1^); Ci (internal CO_2_ concentration—μmol CO_2_ m^−2^ s^−1^); A (CO_2_ assimilation rate—μmol CO_2_ m^−2^ s^−1^); E (transpiration—mmol H_2_O m^−2^ s^−1^); gs (stomatal conductance—mol H2O m^−2^ s^−1^); CEi (instantaneous carboxylation efficiency—[(μmol CO_2_ m^−2^ s^−1^) (μmol CO_2_ m^−2^ s^−1^)^−1^]. FPD (Fruit polar diameter—mm); FED (Fruit equatorial diameter—mm); AFW (Average fruit weight—g per fruit); NFP (Number of fruits per plant); TPP (Total production per plant—g per plant). Averages are followed by standard deviation of the mean (n = 3); means followed by the same letter do not indicate significant differences between treatments according to the Scott–Knott test (*p* > 0.05).

**Table 4:** The analysis of variance for relative water content (RWC), electrolyte leakage (EL), chlorophyll *a* (Chl *a*), chlorophyll *b* (Chl *b*), total chlorophyll (Chl *t*), and carotenoids (Car) at 150 days after transplanting, of sour passion fruit grown under irrigation with water of different levels of electrical conductivity, application methods, and concentrations of ascorbic acid.

Source of Variation	DF	Mean Squares					
		RWC	EL	Chl *a*	Chl *b*	Chl *t*	Car
Application methods (M)	2	1.99 ^ns^	34.67 ^ns^	26,737.19 ^ns^	31,952.81 **	90,892.32 ^ns^	791.90 ^ns^
Ascorbic acid (AsA)	2	0.75 ^ns^	12.72 ^ns^	5039.03 **	54,984.37 **	77,516.81 ^ns^	423.37 ^ns^
Electrical conductivity (ECw)	1	7.05 ^ns^	2904.00 **	65,022.94 **	2331.82 ^ns^	42,727.78 **	245.37 **
Interaction (M × AsA × ECw)	4	3.29 ^ns^	31.69 ^ns^	15,780.78 ^ns^	18,571.82 ^ns^	31,527.80 ^ns^	538.27 ^ns^
Interaction (M × AsA)	4	13.61 ^ns^	27.64 ^ns^	13,309.78 ^ns^	21,702.56 ^ns^	19,106.71 ^ns^	2565.72 ^ns^
Interaction (M × ECw)	2	35.77 ^ns^	20.22 ^ns^	5038.72 ^ns^	1607.52 ^ns^	2205.72 ^ns^	452.72 ^ns^
Interaction (AsA × ECw)	2	339.09 **	15.05 ^ns^	87,585.05 ^ns^	1268.05 ^ns^	29,568.05 ^ns^	421.05 ^ns^
Blocks	2	26.70 ^ns^	44.05 ^ns^	481.17 ^ns^	217.35 ^ns^	284.74 ^ns^	38.27 ^ns^
Residual	34	15.11	51.25	856.39	1432.39	2910.96	27.65
CV (%)		4.68	15.75	12.67	18.32	12.33	10.51

^ns,^ ** respectively non-significant and significant at *p* ≤ 0.01. CV—coefficient of variation, DF—degrees of freedom.

**Table 5:** The analysis of variance for internal CO_2_ concentration (*Ci*), CO_2_ assimilation rate (*A*), transpiration (*E*), stomatal conductance (*gs*), and instantaneous carboxylation efficiency (*CEi*), at 150 days after transplanting, of sour passion fruit grown under irrigation with waters of different electrical conductivity levels, application methods, and ascorbic acid concentrations.

Source of Variation	DF	Mean Squares				
		*Ci*	*A*	*E*	*gs*	*CEi*
Application methods (M)	2	2521.13 ^ns^	1.0 ^ns^	0.16 ^ns^	0.0003 ^ns^	0.0002 ^ns^
Ascorbic acid (AsA)	2	17,420.35 **	2.76 ^ns^	0.05 ^ns^	0.01 **	0.0002 ^ns^
Electrical conductivity (ECw)	1	34,858.96 **	49.06**	1.29 **	0.007 **	0.004 **
Interaction (M × AsA × ECw)	4	3486.60 ^ns^	0.37 ^ns^	0.01 ^ns^	0.0001 ^ns^	0.0002 ^ns^
Interaction (M × AsA)	4	1453.51 ^ns^	3.41 ^ns^	0.11 ^ns^	0.0001 ^ns^	0.0002 ^ns^
Interaction (M × ECw)	2	3141.51 ^ns^	1.01 ^ns^	0.04 ^ns^	0.0002 ^ns^	0.0002 ^ns^
Interaction (AsA × ECw)	2	3486.51 ^ns^	3.66 ^ns^	0.01 ^ns^	0.0002 ^ns^	0.0002 ^ns^
Blocks	2	41.51 ^ns^	2.33 ^ns^	0.01 ^ns^	0.00007 ^ns^	0.0001 ^ns^
Residual	34	471.34	1.89	0.02	0.0001	0.00001
CV (%)		10.72	21.31	18.86	17.25	19.47

^ns,^ ** respectively non-significant and significant at *p* ≤ 0.01. CV—coefficient of variation, DF—degrees of freedom.

**Table 6:** The analysis of variance for initial fluorescence (F_0_), variable fluorescence (Fv), quantum efficiency of photosystem II—(Fv/Fm), maximum fluorescence (Fm), and stem diameter (SD), at 150 days after transplanting, of sour passion fruit grown under irrigation with water of different levels of electrical conductivity, application methods, and concentrations of ascorbic acid.

Source of Variation	DF	Mean Squares				
		F_0_	Fv	Fv/Fm	Fm	SD
Application methods (M)	2	238.9 ^ns^	6414.7 ^ns^	0.000088 ^ns^	6191.90 ^ns^	0.348 ^ns^
Ascorbic acid (AsA)	2	1629 ^ns^	24,939 ^ns^	0.000089 ^ns^	75,752.7 **	4.870 **
Electrical conductivity (ECw)	1	9414 **	42,056 *	0.001645 *	158,437 **	30.31 **
Interaction (M × AsA × ECw)	4	1027 ^ns^	43,150 ^ns^	0.000664 ^ns^	2238.27 ^ns^	0.905 ^ns^
Interaction (M × AsA)	4	514.4 ^ns^	2198.4 ^ns^	0.00019 ^ns^	19,700.7 **	1.308 ^ns^
Interaction (M × ECw)	2	1094 ^ns^	3404.7 ^ns^	0.000252 ^ns^	8583.72 ^ns^	1.054 ^ns^
Interaction (AsA × ECw)	2	2086 ^ns^	3230.2 ^ns^	0.0000006 ^ns^	11,268.05 ^ns^	1.499 *
Blocks	2	225.6	11,128	0.00083	2238.27	0.082
Residual	34	601.7	6975.09	0.00040	3343.65	0.375
CV (%)		5.10	5.70	2.58	3.05	4.02

^ns,^ *, ** respectively non-significant and significant at *p* ≤ 0.05 and *p* ≤ 0.01. CV—coefficient of variation, DF—degrees of freedom.

**Table 7:** The analysis of variance for fruit polar diameter (FPD), fruit equatorial diameter (FED), average fruit weight (AFW), number of fruits per plant (NFP), total production per plant (TPP), and pulp volume (PV), at 180 days after transplanting, of sour passion fruit grown under irrigation with water of different levels of electrical conductivity, application methods, and concentrations of ascorbic acid.

Source of Variation	DF	Mean Squares					
		FPD	FED	AFW	NFP	TPP	PV
Application methods (M)	2	190.39 **	583.18 **	5576.99 **	6.01 ^ns^	40,933.38 ^ns^	288.06 **
Ascorbic acid (AsA)	2	133.62 **	289.99 ^ns^	3292.81 **	4.57 ^ns^	207,195.71 ^ns^	1362 **
Electrical conductivity (ECw)	1	10.17 ^ns^	189.01 ^ns^	6.11 ^ns^	303.41 **	7,855,116.07 **	46.29 ^ns^
Interaction (M × AsA × ECw)	4	17.93 ^ns^	369.24 ^ns^	722.61 ^ns^	35.91 ^ns^	225,935.91 ^ns^	71.49 ^ns^
Interaction (M × AsA)	4	89.57 ^ns^	167.07 ^ns^	871.78 ^ns^	17.79 ^ns^	59,213.39 ^ns^	382.37 **
Interaction (M × ECw)	2	27.66 ^ns^	284.65 ^ns^	482.68 ^ns^	18.37 ^ns^	238,400.23 ^ns^	323.97 ^ns^
Interaction (AsA × ECw)	2	12.07 ^ns^	98.75 ^ns^	939.10 ^ns^	18.21 ^ns^	150,123.95 ^ns^	314.75 **
Blocks	2	23.28 ^ns^	86.47 ^ns^	28.55 ^ns^	0.90 ^ns^	18,694.25 ^ns^	188.56
Residual	34	28.07	120.27	225.01	15.69	126,741.20	54.242
CV (%)		7.47	10.63	14.33	19.06	14.26	12.77

^ns,^ ** respectively non-significant and significant at *p* ≤ 0.01. CV—coefficient of variation, DF—degrees of freedom.

**Table 8:** Description of the treatments used.

ECw	Ascorbic Acid Application Methods (M)			Concentration
	M1 (Soaking)	M2 (Spraying)	M3 (Soaking + Spraying)	
S1 (0.8 dS m^−1^)	S1M1C1	S1M2C1	S1M3C1	C1—0 mM
	S1M1C2	S1M2C2	S1M3C2	C2—0.8 mM
	S1M1C3	S1M2C3	S1M3C3	C3—1.6 mM
S2 (3.8 dS m^−1^)	S2M1C1	S2M2C1	S2M3C1	C1—0 mM
	S2M1C2	S2M2C2	S2M3C2	C2—0.8 mM
	S2M1C3	S2M2C3	S2M3C3	C3—1.6 mM

ECw—electrical conductivity of irrigation water.

## Data Availability

Data are contained within the article. No supplemental data are provided.
